# Molecular epidemiology of acute hemorrhagic conjunctivitis caused by coxsackie A type 24 variant in China, 2004–2014

**DOI:** 10.1038/srep45202

**Published:** 2017-03-23

**Authors:** Li Zhang, Na Zhao, Xiaodan Huang, Xiuming Jin, Xingyi Geng, Ta-Chien Chan, Shelan Liu

**Affiliations:** 1 Eye Center of the Second Affiliated Hospital, Medical School of Zhejiang University, Hangzhou, Zhejiang Province, China; 2 National Research Center for Wildlife Borne Diseases, Key Lab of Animal Ecology and Conservation Biology, Institute of Zoology, Chinese Academy of Sciences, Beijing, China; 3 Emergency Offices, Jinan Centre for Disease Control and Prevention, Jinan, Shandong Province, China; 4 Center for Geographic Information Science, Research Center for Humanities and Social Sciences, Academia Sinica, Taipei, Taiwan; 5 Department of Infectious Diseases, Zhejiang Provincial Center for Disease Control and Prevention, Hangzhou, Zhejiang Province, China

## Abstract

To understand control interventions, the molecular epidemiology of acute hemorrhagic conjunctivitis (AHC) was investigated from 2004 to 2014.A total of 613,485 AHC cases (annualized cases 55,771) with two deaths were included. Our findings showed that AHC was reported in all provinces, predominantly in Southern and Eastern China. The incidence rates were highest in 2007 (5.65/100,000) and 2010 (21.78/100,000) respectively. A clear seasonal pattern was identified with a peak from August to October. AHC cases occurred in all age groups; however, five to 14 years was the predominant group [23.06%, 133, 510/578,909]. The median age was 24 years (one month~97 years). The median duration from onset to diagnosis was 1.5 days, and there was no difference between the <15, 15~60 and >60-year-old patients [*p* = 0.0653]. The phylogenetic analysis of 100 nonstructural proteins (3C) and 84 structural proteins (VP1) revealed that AHC outbreaks were caused by Coxsackievirus A24 variant. Genotypes G4-c5a, G4-c5b, and G4-c3 co-circulated with both temporal and geographical overlaps. In conclusion, despite the overall steady decline in the number of AHC cases since the peak in 2010, it still remains a serious public health problem in Southern and Eastern China that targets on the school aged children under 15 years old.

Acute hemorrhagic conjunctivitis (AHC) is an epidemic form of highly contagious conjunctivitis that is predominantly caused by the Coxsackievirus A24 variant (CA24v), enterovirus 70 (EV70) or adenovirus^1^,^2^. Recently, CA24v has been implicated as a major causative agent of AHC outbreaks in several countries^3^,^4^,^5^,^6^. The majority of patients experience a self-limited illness, typically including the sudden onset of painful, swollen, red eyes with conjunctival hemorrhaging and excessive tearing; however, a small proportion of patients rapidly develop systemic symptoms^1^,^4^, such as fever, fatigue and limb pain. Generally, severe complications and fatal infections have been rare^7^,^8^,^9^,^10^,^11^.

Since the outbreak of AHC caused by CA24v was first recorded in Singapore in 1970^12^, it has been reported throughout the world, predominantly in tropical and subtropical regions. CA24v has spread extremely rapidly in Asia, such as in Thailand, South Korea and Japan^3^,^10^,^13^,^14^,^15^. In 2002, the largest AHC outbreak due to CA24v was identified in South Korea with over one million cases^12^. The first outbreak of AHC in China was reported in Hong Kong in 1971, and a nationwide AHC epidemic caused by CA24v was reported in 2007^13^,^16^ with a total of 74,263 AHC cases. From 2007 to 2010, additional CA24v-associated AHC outbreaks were reported in Yunnan, Guangdong and Jiangsu Provinces as well as in Taiwan^4^,^13^,^17^,^18^,^19^. AHC could affect 50% of the population in communities within one or two months due to super human-to-human transmission^7^,^11^,^20^. Thus, AHC outbreaks can disrupt the local economy, and they require substantial healthcare resources in local regions.

AHC outbreaks have become a severe public health issue in China, particularly in school-aged children^1^. To manage the epidemiological status of AHC, in 2003, China’s government launched the National Disease Supervision Information Management System (NDSIMS) for notification of infectious diseases. AHC belongs to the Class C notifiable infectious disease category in China. AHC cases diagnosed by physicians should be registered through the public health surveillance program within 24 hours of a diagnosis^13^,^19^. For this study, the notifiable surveillance data from 2004 to 2014 in China was used to analyze the molecular epidemiological features in the largest sample size with the aims to identify the specificities of the high-risk areas, populations and seasons and the clinical severity as well as to identify the etiological agent that caused the outbreaks. This research will assist in planning and developing resources that can decrease the transmission of this highly contagious disease and will increase government and public awareness regarding the potential risk of AHC and the means to control and prevent AHC outbreaks in the future.

## Results

### Epidemic features

**Figure 1:**
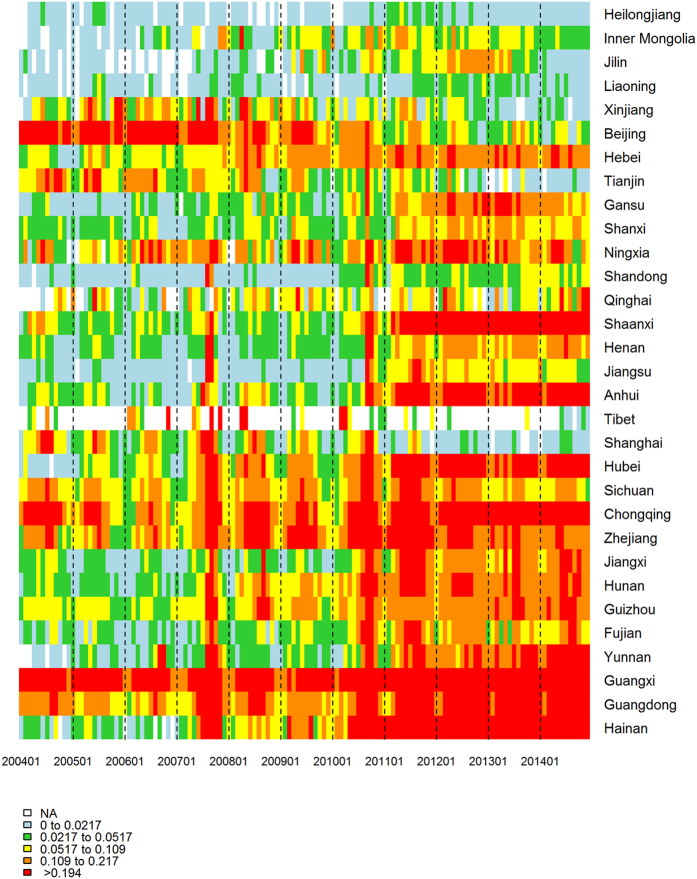
Heatmap of AHC surveillance data from 2004 to 2014 by Chinese province. The provinces were ordered by latitude from northernmost (top) to southernmost (bottom). Time series of weekly AHC cases standardized by the incidence of annual cases. Notes: A heat map created using the gplot package in R software (https://cran.r-project.org/web/packages/gplots/index.html).

**Figure 2:**
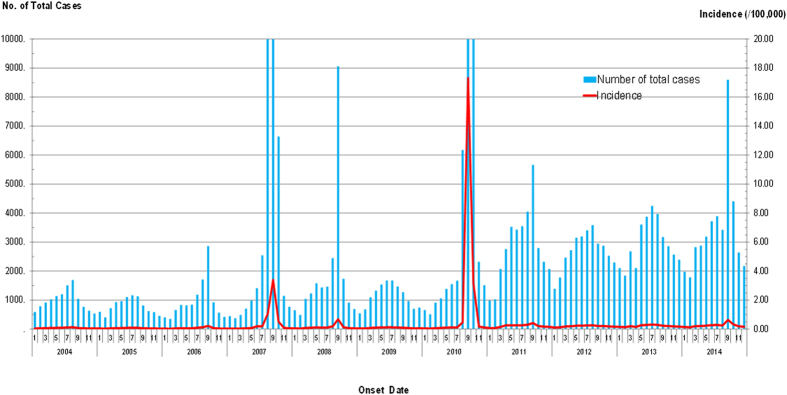
Timeline by illness onset month of AHC cases from 2004 to 2014 in China. There was an obvious seasonal fluctuation of AHC with a single peak.

From 2004 to 2014, 613,485 AHC clinical diagnosis cases were reported to the NDSIMS with a yearly average of 55,771. The lowest incidence was identified in 2005 with 0.73/100,000 (n = 9,493) cases. The highest incidence was observed in 2010 (21.78/100,000, n = 290,767). Since then, the incidence rate has gradually decreased to a fixed level in 2014 (3.06/100,000, n = 41,514) (Figs 1 and 2).

**Figure 3:**
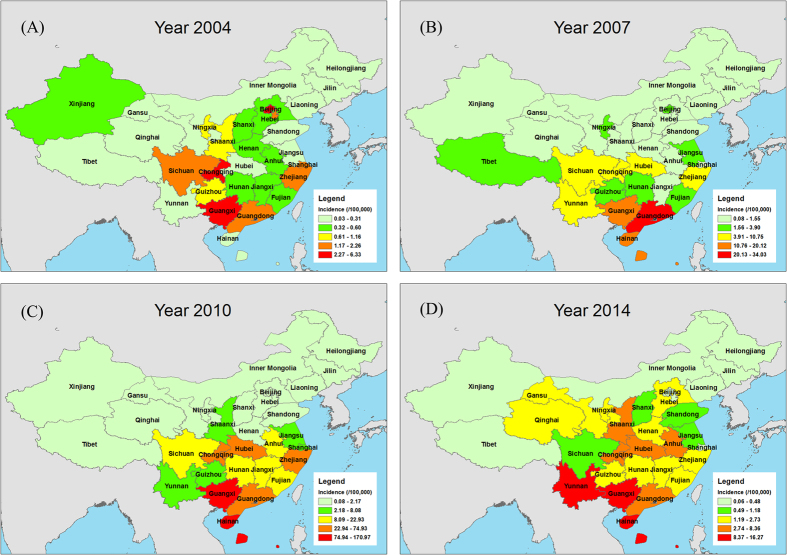
Geographic distributions of AHC cases from 2004 to 2014 in China. (**A**) for 2004; (**B**) for 2007; (**C**) for 2010; (**D**) for 2014. The numbers and colors in the legend represent the incidence of AHC cases. These maps were generated using the software ArcGIS (ArcMap, version10.2; ESRI Inc., Redlands, CA, USA, https://www.qgis.org/en/site/forusers/download.html). The world (http://openstreetmapdata.com/data/land-polygons) and China (https://mapzen.com/data/borders/) basemaps we used are all from OpenStreetMap. The cartography in the OpenStreetMap map tiles is licensed under CC BY-SA (www.openstreetmap.org/copyright). The license terms can be found on the following link: http://creativecommons.org/licenses/by-sa/2.0/.

**Figure 4:**
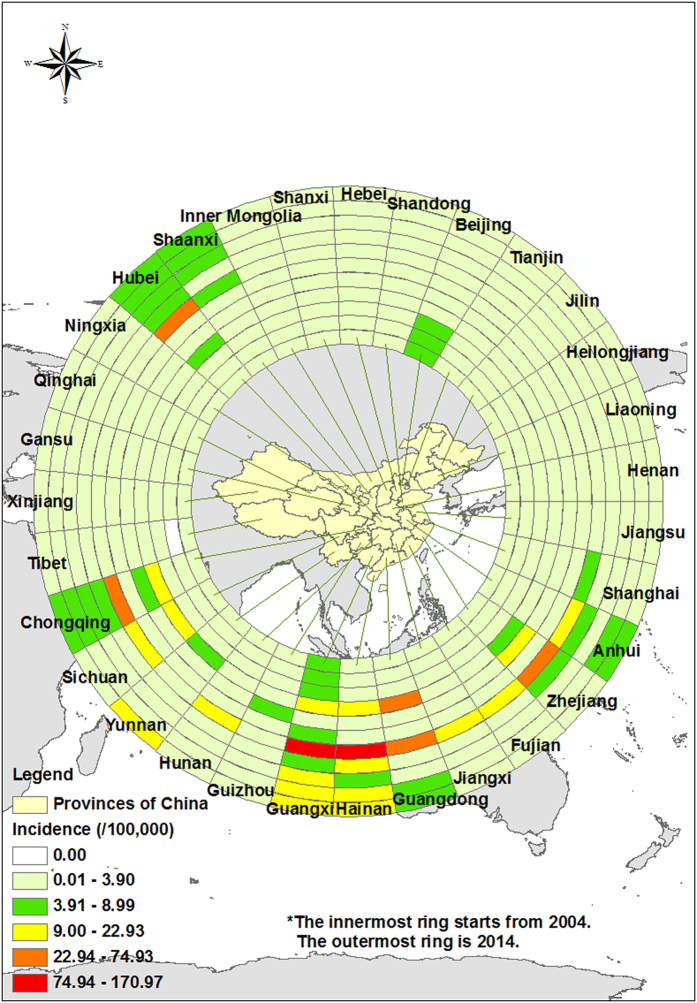
Ring map of AHC cases from 2004-2014 standardized by the incidence of annual cases in Chinese provinces. Notes: The ring map toolbox in ArcGIS (Generated by ArcGIS 10.2, URL: http://www.esri.com/software/arcgis/arcgis-for-desktop, ESRI Inc., Redlands, CA, USA) was developed by the co-author Ta-Chien Chan. The toolbox was freely available from the website (http://www.esri.com/esri-news/arcuser/fall-2013/looking-at-temporal-changes). The world (http://openstreetmapdata.com/data/land-polygons) and China (https://mapzen.com/data/borders/) basemaps we used are all from OpenStreetMap. The cartography in the OpenStreetMap map tiles is licensed under CC BY-SA (www.openstreetmap.org/copyright). The license terms can be found on the following link: http://creativecommons.org/licenses/by-sa/2.0/.

Regarding geographical distribution, all 31 provinces/areas reported AHC cases with a predominance in the Eastern and Southern regions of China, namely in Guangxi, Guangdong and Zhejiang provinces, accounting for 48.71% (298, 838/613,485) of cases. In contrast, the Western and Northeastern provinces reported the lowest number of cases, which only accounted for 1.14% of total cases (6,984/613,485) (Figs 3A–D and 4).

Based on the incidence rate, three different epidemic levels were identified, including the highest level (L1, >4/100,000 cases) in 8 provinces (Guangxi, Hainan, Yunnan, etc.); the middle level (L2, ranging from 1~3.99/100,000 cases) in 12 provinces (Hunan, Guizhou, Hebei, etc.); and the lowest level (L3, below 1/100,000 cases) in the other 11 provinces ((Fig. 3A–D).

Regarding the seasonal pattern, AHC occurred throughout the year; however, most cases were reported from August to October (72.72% of total cases, 421,438/577,948) and usually peaked in August, as shown in Fig. 2.

**Figure 5:**
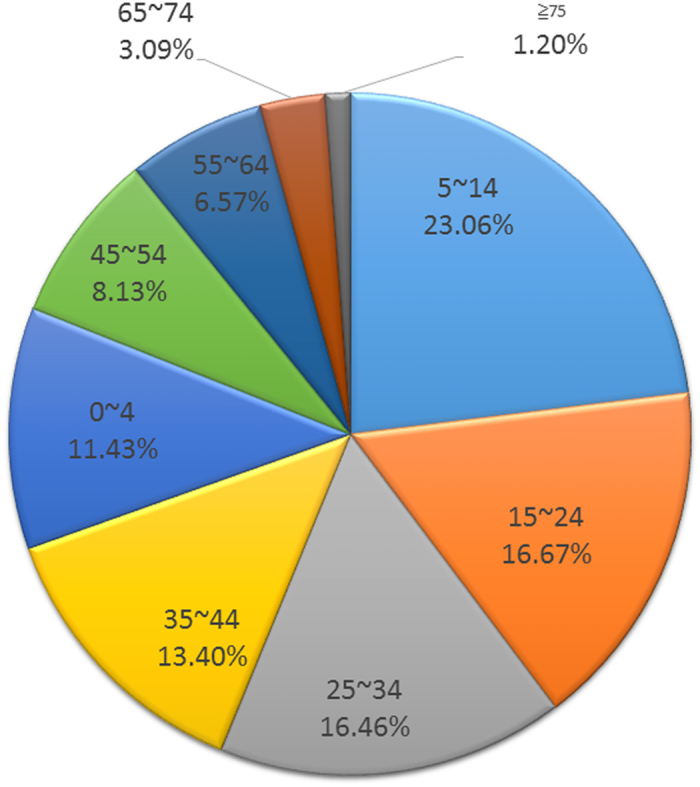
Age distribution of AHC cases in China from 2004–2014.

For the population distribution, AHC occurred in all age groups, which ranged from one month to 97 years (48,591 available cases). The median age was 24 years old. The highest number of cases occurred in the 5~14 age group, accounting for 23.06% (133,510/578,909) of total cases (Fig. 5). The incidence of AHC was 1.4-fold higher in the male group than in the female group.

In terms of occupational distribution, the major population affected was students, accounting for 28.36% (13,782 cases). The second and third highest populations affected were farmers and kindergarten children, with 16.62% (8,075) and 11.90% (5,780) of cases, respectively (Supplementary Fig. S1).

### Clinical severity

Only two cases in 613,485 overall cases reported in China resulted in death. The mortality rate was 0.0003%. The median days from illness onset to diagnosis was 1.5 days (range: 0–16 days); however, no significant difference was observed in the onset-to-diagnosis intervals among the three groups [1.0 days in children (<15 years) vs. 2.0 days in adults (15~60) vs. 2.0 days in the elderly (>60 years), *p* = 0.0653] (Supplementary Fig. S2). In addition, the median number of days from onset to diagnosis in the female cases was not significantly different from the male cases (1.5 days for each, *p* = 0.0900) (Supplementary Fig. S3).

### Molecular evolution of 3C region from the human CA24v strain

**Figure 6:**
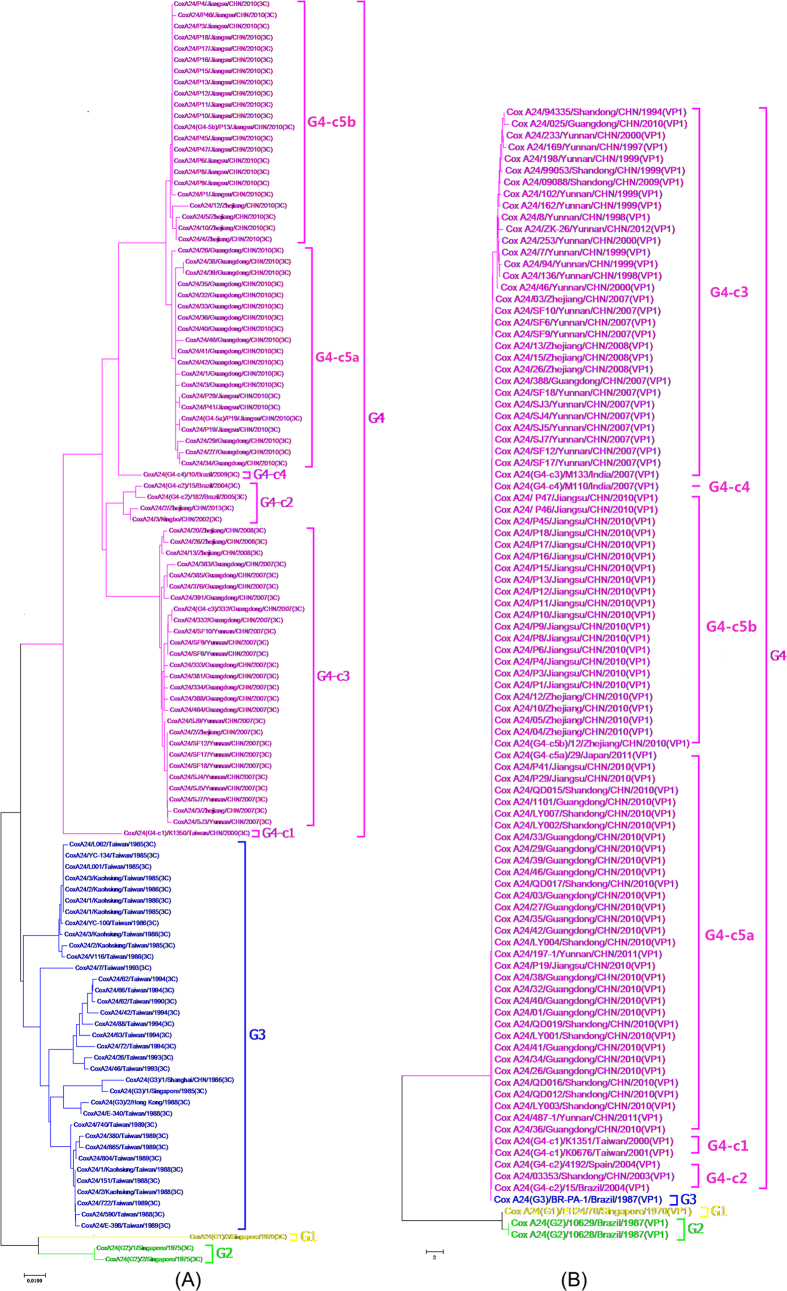
Phylogenetic analyses based on the representative strains of the nonstructural protein 3C and the VP1 gene of Cox A24 viruses in China from 1985–2014. (**A**) for 3C (n = 113); (**B**) for VP1 gene (n = 119).

For the present study, 100 nonstructural proteins (3C) [a 510 bp fragment] were selected as the representatives of CA24v strains from China. The findings indicated that the intra-sequence variation in the same subtypes was 3.4~5.5%; however, the heterogeneity increased 8~9% among the different subtypes (Fig. 6A). The phylogenetic analysis showed that all sequences were separated into two clades: G3 and G4 (G4-c5a, G4-c5b, G4-c3, and G4-c2).

For the genotype percentages, genotype G3 accounted for 33.00%, genotype G4-c3 for 25.00%, genotype G4-5b for 21.00%, genotype G4-5a for 19.00%, and genotype G4-c2 for 2.00%. In general, genotypes G4-5a, G4-5b, and G4-c3 were the major strains from 2010 to 2014 in China.

In terms of the geographical distribution of the genotypes, G4-c3 was the predominant subtype in Southwestern and Southern China. G4-5b mainly circulated in Eastern China, and the G4-5a subtype was the major subtype identified in Southern and Eastern China. G4-c2 was only found in the Zhejiang Province, which is located in Eastern China.

### Molecular evolution of the 3′VP1 region from the human CA24v strain

A total of 84 structure protein VP1 [a 235 bp fragment] was selected as the representatives of CA24v strains. The variation in the 3′VP1 region was 0~14.0% among these sequences. The phylogenetic tree showed that all strains clustered in one large clade: G4 (G4-c5a, G4-c5b, G4-c3, and G4-c2) (Fig. 6B).

The predominant subtypes were G4-5a (32, 38.10%), followed by G4-c3 (30, 35.71%), G4-5b (21, 25.00%), and G4-c2 (1, 1.19%). G4-5a and G4-5b were the predominant strains after 2010.

For the geographical distribution of the genotypes, G4-5b predominantly circulated in Eastern China. G4-5a was mainly identified in Southern China; however, G4-c3 was most often found in Southeast China.

## Discussion

There were four periods of global AHC outbreaks. The first was the emerging period in 1970s, which was recognized by limited transmission. Second, the virulent period during the 1980s was characterized by several outbreaks in Northeast Asia, the Western Hemisphere and North Africa. Third, the silent period during the 1990s was characterized by only a few outbreaks reported in East Asia from 1993–1994. Finally, the reemerging period during the 2000s has been identified by extremely rapid transmission throughout the world^3^. China was identified as one of the most affected countries, and epidemics of AHC caused by CA24v reappeared in China beginning in 2007^13^. In this study, a large sample of more than 600,000 AHC cases reported to the National Enhanced Surveillance System from 2004 to 2014 in China was utilized, and it provides the first comprehensive account of the epidemiological and clinical features and genotype distribution of the disease in the past eleven years.

This study included 31 climatologically diverse provinces, and the results suggest that AHC cases tend to occur throughout the country, with the periodicity of epidemics varying based on latitude. Outbreaks of CA24v tend to occur in the provinces/areas between latitude 40° North and 15° South^11^. The major epidemic areas were in the east and south of China based on the national routine surveillance, and significantly higher numbers of cases were identified in Guangxi, Guangdong and Yunnan Provinces. Since 2010, several large epidemics of AHC outbreaks have occurred that were also reported in the south and east of China, including Guangdong, Yunnan, Jiangsu and Zhejiang Provinces^13^,^17^,^18^,^19^,^21^,^22^, and the outbreaks were caused by CA24v^13^. In contrast, the west and the north of China were the areas with low epidemic rates. The differences in geographical distribution were related to three major factors. First, the population in the heavy infection areas was dense compared to the low epidemic areas, and the population in Eastern China was estimated at 50 to 90 million inhabitants, which included an increasing number of school aged children. Because the children were not vaccinated against this strain and have no or low level CA24v antibody, they may have been a possible source of this re-emergence^23^. Secondly, it is conceivable that the climate of Eastern and Southern China may be unfavorable for the dissemination of this enterovirus^19^. Third, the surveillance capability in Eastern and Southern China (developed areas) was much higher and more sensitive than the provinces of China in the west and north (developing areas)^13^,^17^,^18^,^19^.

The epidemiology of AHC human infections has been characterized by two peaks in the past ten years. The first was in the summer of 2007 when 74,263 cases were recorded, representing an increase of 540.58% compared with the 11,593 cases in 2006^19^. The second peak began in late June 2010 and peaked that July, with a total of 290,767 cases reported nationwide that year (near 4-fold the number in 2007). Three factors might have contributed to the differences between the two peaks. First, the national surveillance systems for AHC cases were enhanced, and there was a considerable increase in the number of specimens tested throughout China since 2007^17^,^18^. Second, outbreaks of AHC reemerged with CA24v as a major pathogen in 2010^13^,^19^ worldwide, and most people were not immune to this new virus. Third, outbreaks of AHC usually occurred periodically over two to three consecutive years. One reason this occurred is that the neutralizing antibody to both CA24v and EV70 does not last more than seven years, and the other reason is that there is no cross reaction of neutralizing antibodies between EV70 and CA24v^24^.

The large-scale data suggested that AHC cases have a single peak in the summer in different provinces of China. AHC seasonal patterns in China are similar to other Asian countries, such as Thailand, Japan, Singapore, and South Korea^8^,^25^. AHC outbreaks demonstrated a cyclical and seasonal pattern, especially during the rainy season, every two-three years in these countries^26^,^27^. One contributor is that the climate during this period is characterized by high temperatures and frequent rainfall, which may be two climatic factors involved in the rapid spread of CA24v strains^26^. A more important contributing factor is that the autumn season begins at the end of August to early September, which is when school-aged children are in crowded classrooms and when there is extensive contact between family members and students.

The mean age of AHC patients was 24 years, and a large proportion of patients was comprised of primary and junior school students (5~14 years old) among all age groups^23^. The lowest proportion of patients was the elderly (>75 years old). A lack of infection or the low incidence rate for infants younger than six months was most likely due to protection by maternal antibodies. In general, the features of this epidemic has a tendency to affect younger patients. The observed age profile of AHC was in agreement with reports from the Pakistani, Japanese, and South Korean epidemics, for example^15^,^23^,^28^. Similarly, Wu *et al*. reported that 23.9% of cases were students, followed by factory workers (22.8%) and children in kindergarten (16.8%), in the Chinese epidemic^13^. In this study, AHC outbreaks were mostly found to affect students, farmers, and kindergarten children^17^,^23^,^29^. The reasons for the predominance in younger students are not fully understood; however, school students could be affected due to unhygienic habits and AHC spreading rapidly in schools. This suggested person-to-person transmission among school-aged children accounted for the widespread epidemic in China. Spending time in closed, crowded environments has been identified as a possible cause for the predominance in children^19^. School-aged children in rural areas had the highest risk of infection^7^. Thus, to control outbreaks of AHC, prevention methods should target these high-risk students. School exclusion might be effective in controlling AHC epidemics^23^.

CA24v has been implicated as a major causative agent of AHC outbreaks in many countries^3^. Several epidemics of AHC caused by CA24v have been reported in the last decade in Asia. CA24v has been classified into four genotypes based on a phylogenetic analysis of the 3Cpro and VP1 region of CA24v^30^. Major global epidemics have been attributed to four genotypes of CA24v strains, which are G1, G2, G3 and G4. Genotype 1 (G1) includes early strains isolated in Singapore and Hong Kong in 1970-1971^31^,^32^,^33^,^34^. G2 includes Singapore and Thailand’s isolated strains obtained in 1975^30^,^35^. G3 includes six clusters of strains isolated beginning in 1985 in Asia, Africa and France^30^. G4 includes six clusters of strains that have been identified to date^3^,^36^. This study demonstrated that CA24v was the causative agent of AHC outbreak in China during 2004 to 2014. G4-c3, G4-c5a and c5b were the predominant strains in China from 2004 to 2014. The three genotypes co-evolved and co-circulated in Eastern and Southern China. These results of the study are similar to other reports, which indicated that the CA24v strains that caused the outbreaks belonged to the G4 group in Zhejiang and Guangdong Provinces^17^,^18^; however, the genetic diversity, adaptation and fitness of CA24v and the lack of a cross reaction for neutralizing antibodies between different genotypes of CA24v in China^23^ will lead to outbreaks in the future. The same strain of CV-A24v was implicated in the AHC outbreaks in both China and Egypt in 2010, which belonged to G4, but they were different from the outbreaks in Africa in 2004 and in Korea in 2002 (G3)^14^. These findings suggest that the present CA24v strains causing AHC in these regions are genetically related and that these AHC strains emerged around 2010. The reason for this re-emergence of CA24v AHC in China requires further investigation, such as a serological survey of the total population and a molecular-based investigation.

Several limitations should be noted. First, as for any common, self-limiting illness, surveillance only captures the tip of the clinical iceberg, while most cases go undetected because the patient’s condition is asymptomatic, because the patient does not seek formal care, or because he/she is not diagnosed. Second, access to and provision of healthcare as well as technical capacity varies between and sometimes within provinces, and there is no formal quality assurance or systematic audit for AHC surveillance. Third, data provided during the first year of collection are likely less reliable than those from more recent years. Fourth, an investigation of family members and students could not be conducted due to recall biases, so epidemic patterns between intra-family and intra-school transmissions could not be determined. This leads to population mixing, and this research focused on the effects of the infection on school pupils.

In conclusion, substantial AHC illnesses resulted in rare severe cases caused by the CA24v strain (Genotypes G4-c5a, 5b, and G4-c3) from 2004 to 2014, which is notable despite the overall steady decline of cases since the peak in 2010. The occurrence of AHC cases was mainly found in Eastern and Southern China, with one single peak in August each year. AHC cases disproportionately affected school-aged students from five to 14 years old. The disease distribution did not change over the years.

Overall, the study provides robust, population-based national data that can be used to prioritize the high-risk target population and to add more public health resources on the heavy affecting areas in the peaked seasons.

Effective interventions should be implemented to control outbreaks of AHC as soon as possible due to its extremely contagious nature, and prevention methods should target groups at the highest risk and schools in high-risk areas (school-aged children, persons living in crowded urban areas and household contact of infected persons) including encouraging careful and frequent handwashing and avoiding sharing towels, bedding, makeup and other personal items with persons with conjunctivitis. Information should also be disseminated to the public after the first report of AHC in the area. In addition, continuous, adequate and functioning surveillance systems are required for early discovery, reporting and diagnosis, particularly in asymptomatically infected students in schools.

## Methods

### Ethics statement

This study was approved by the Ethics Committee of the Zhejiang Provincial Center for Disease Control and Prevention, and the committee members reviewed research protocols and related materials and methods. The protocol review assessed the ethics of the research and its methods, which promoted fully informed and voluntary participation. All methods in this study were confirmed to be performed in accordance with the Chinese relevant guidelines and regulations with surveillance research by the Zhejiang Ethical Review Board. All CA24v sequences data were obtained from publicly available data sources.

### The informed consent statement

It was confirmed that public health doctors obtained written informed consent for participation from all outbreak subjects or their legal guardians (n = 98,591) from 2004 to 2014 before conducting the field invention and sample collection. For the non-outbreak cases, the clinical doctors obtained permission from the patients when they began consultation and receive the treatment in hospitals. All data were supplied and analyzed in an anonymous format, with no personal identifying or sensitive information provided to researchers.

### Case definition

According to the diagnosis and treatment programs of AHC issued by the National Health and Family Planning Commission of the People’s Republic of China (http://www.chinacdc.cn/jkzt/crb/bl/jxcxxjmy/jszl_2255/200709/t20070918_24797.html), an AHC case is defined as the onset of redness, tearing, swelling, itching and/or burning around one or both eyes for at least a 1-day duration within the preceding 8 weeks.

### AHC cases source

All AHC case information used in this study was collected from NDSIMS (http://www.phsciencedata.cn/Share) in China from 2004 to 2014.

### The sequences source for non-structure 3C and VP1

The representative non-structure 3C and VP1 sequences from the CA24v strains collected were retrieved from GenBank (https://www.ncbi.nlm.nih.gov/nuccore/?term=CA24v). There was a total of 113 non-structure 3C sequences (including 13 references) from 1985–2014 and 96 VP1 sequences from 1985–2014 (including 12 references). The sequence accession numbers and genotypes are listed in Supplementary Tables S1 and S2. The sequences were selected based on the following criteria: first, sequences generated from low- and high-virulence/pathogenic strains; second, sequences responsible for outbreaks; third, sequences from different serotypes in different years; and fourth, sequences from high-circulating and low-circulating areas.

### Spatial analyses

ArcGIS (ArcMap, version10.2; ESRI Inc., Redlands, CA, USA, https://www.qgis.org/en/site/forusers/download.html) was used for the spatial analysis, and a map was generated for AHC cases across all provinces in China. The world (http://openstreetmapdata.com/data/land-polygons) and China (https://mapzen.com/data/borders/) basemaps we used are all from OpenStreetMap (www.openstreetmap.org/copyright).

### Seasonality analyses

To quantify AHC seasonal patterns by province, heat maps of the proportion of AHC cases identified in each month were created using the gplot package in R software (https://cran.r-project.org/web/packages/gplots/index.html) and using the ring map toolbox in ArcGIS 10.2.

### Population analyses

SPSS (v17.0, SPSS Inc, Chicago, IL, USA, http://www.spss.com.hk/corpinfo/index.htm) was used to analyze the population distribution, including the median age, sex, and occupation. The annual population denominators during the study period were obtained from the National Bureau of Statistics of China.

### Molecular serotyping of CA24v

The comparison of sequences was performed using the BLAST programs (www.ncbi.gov/blast/) from NCBI. If the pairwise identity scores were 75% with respect to any particular enterovirus sequence in GenBank, then it was identified as a homologous serotype. To identify respective divergences and to infer the genetic relationships among the isolates, a sequence analysis for VP1 and 3C sequences was performed using MEGA7.0 software. The phylogeny was analyzed, and the resulting trees were constructed using a neighbor-joining method of reconstruct phylogeny.

### Statistical analysis

An analysis of variance (F test) was applied to the measured data. Chi-square tests (*x*^2^) were used to compare the distributions of the different variables of the qualitative measurements. All statistical analyses were performed using SPSS (v17.0, SPSS Inc, Chicago, IL, USA). For all analyses, probabilities were 2-tailed, and a p-value of < 0.05 was considered statistically significant.

## Additional Information

**How to cite this article:** Zhang, L. *et al*. Molecular epidemiology of acute hemorrhagic conjunctivitis caused by coxsackie A type 24 variant in China, 2004-2014. *Sci. Rep.* **7**, 45202; doi: 10.1038/srep45202 (2017).

**Publisher's note:** Springer Nature remains neutral with regard to jurisdictional claims in published maps and institutional affiliations.

## Supplementary Material

Supplementary Information
