# Supplementary material for: Molecular epidemiology of acute hemorrhagic conjunctivitis caused by coxsackie A type 24 variant in China, 2004–2014
Source: Sci Rep. 2017 Mar 23;7:45202. doi: 10.1038/srep45202 (PMC5362916; doi:10.1038/srep45202)
Supplement: Supplementary Information [file srep45202-s1.pdf]

Supplementary information

Molecular epidemiology of acute hemorrhagic conjunctivitis caused by coxsackie A type 24 variant in China, 2004-2014

Li Zhang[1], Na Zhao[2], Xiaodan Huang[1], Xiuming Jin[1], Xingyi Geng[3], Ta-Chien Chan[4*], Shelan Liu[5*]

1. Eye Center of the Second Affiliated Hospital, Medical School of Zhejiang University, Hangzhou, Zhejiang Province, China;
2. National Research Center for Wildlife Borne Diseases, Key Lab of Animal Ecology and Conservation Biology, Institute of Zoology, Chinese Academy of Sciences, Beijing, China;
3. Emergency Offices, Jinan Centre for Disease Control and Prevention, Jinan, Shandong Province, China;
4. Center for Geographic Information Science, Research Center for Humanities and Social Science, Academia Sinica, Taipei, Taiwan;
5. Department of Infectious Diseases, Zhejiang Provincial Center for Disease Control and Prevention, Hangzhou, Zhejiang Province, China.

* **Corresponding author**

Dr. Shelan Liu, Ph.D., Department of Infectious Diseases, Zhejiang Provincial Centre for Disease Control and Prevention, 3399 Binsheng Road, Binjiang District, Hangzhou, Zhejiang Province, China (310051). E-mail: liushelan@126.com. Tel: (+86) 571-87115137; Fax: (+86) 571- 87115130.

Dr. Ta-Chien Chan, Ph.D., Research Center for Humanities and Social Sciences, Academia Sinica, Nankang, Taipei, Taiwan. Email: dachianpig@gmail.com.

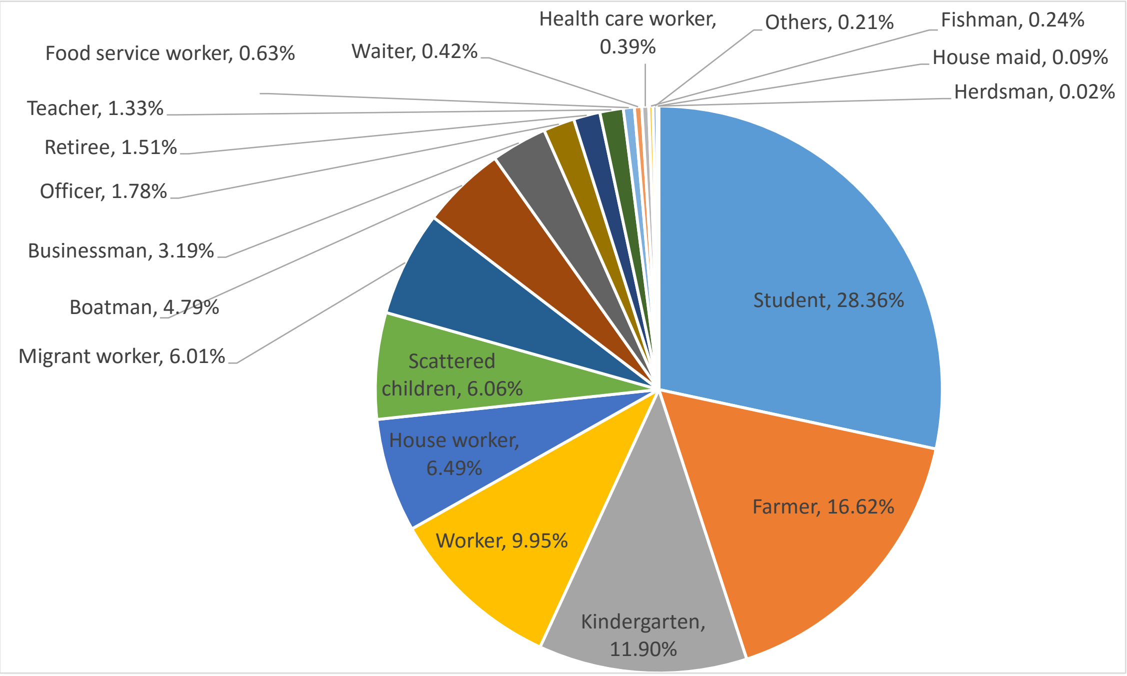

Supplementary Figure 1. Occupational distribution of AHC cases in China during 2004-2014 (n=48,591)

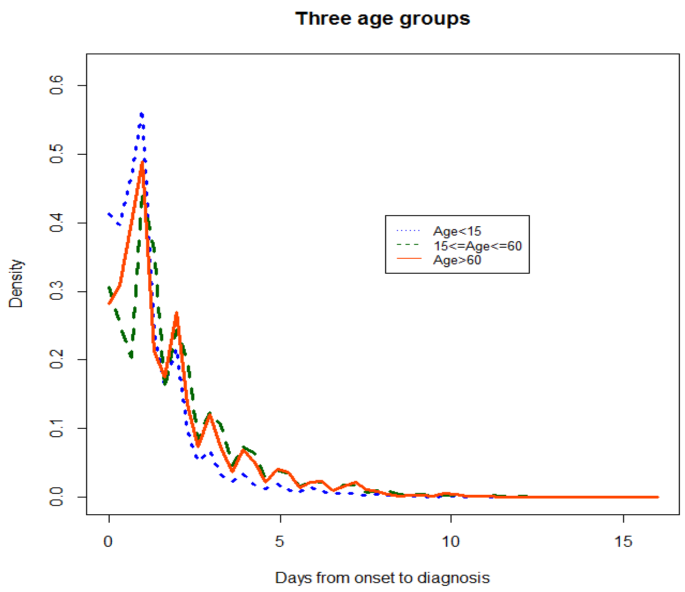

Supplementary Figure 2. The median days from illness onset to be diagnosed in the children (<15 years, adults (15~60) and old people (>60 years old) of AHC cases from China during 2004-2014 (n=48,591)

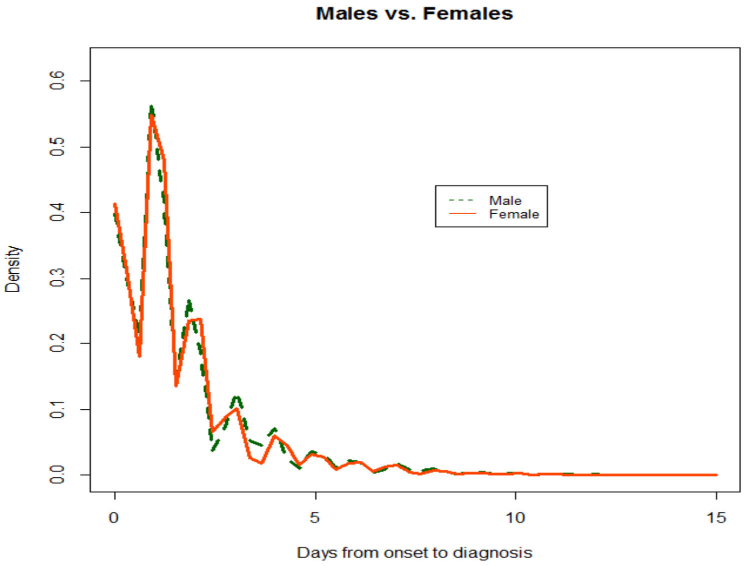

Supplementary Figure 3. The median days from illness onset to be diagnosed in male and female AHC cases from China during 2004-2014 (n=48,591)

Supplementary table 1. Accession numbers of non-structural protein (3C) gene from Human coxsackievirus A24 isolate used in this study isolated in China (N=113)

Country	Area	Collect year	Accession number	Strain Name	Genotype	Notes
Singapore	Unknown	1970	D10295	Cox A24(G1)/3/Singapore/1970 (3C)	G1	Reference strain
Singapore	Unknown	1975	D10298	Cox A24(G2)/1/Singapore/1975 (3C)	G2	Reference strain
Singapore	Unknown	1975	D10299	Cox A24(G2)/2/Singapore/1975 (3C)	G2	Reference strain
China	Taiwan	1985	D10306	Cox A24/3/Kaohsiung/Taiwan/1985(3C)	G3	
China	Taiwan	1985	D10305	Cox A24/2/Kaohsiung/Taiwan/1985(3C)	G3	
China	Taiwan	1985	D10304	Cox A24/1/Kaohsiung/Taiwan/1985(3C)	G3	
China	Taiwan	1985	D13270	Cox A24/L062/Taiwan/1985(3C)	G3	
China	Taiwan	1985	D13269	Cox A24/L001/Taiwan/1985(3C)	G3	
China	Taiwan	1985	D13271	Cox A24/YC-134/Taiwan/1985(3C)	G3	
Singapore	Unknown	1985	D10301	Cox A24(G3)/1/Singapore/1985 (3C)	G3	Reference strain
China	Taiwan	1986	D10310	Cox A24/3/Kaohsiung/Taiwan/1986(3C)	G3	
China	Taiwan	1986	D10309	Cox A24/1/Kaohsiung/Taiwan/1986(3C)	G3	
China	Taiwan	1986	D10308	Cox A24/2/Kaohsiung/Taiwan/1986(3C)	G3	
China	Taiwan	1986	D13274	Cox A24/YC-100/Taiwan/1986(3C)	G3	
China	Taiwan	1986	D13272	Cox A24/V116/Taiwan/1986(3C)	G3	
China	Shanghai	1986	D10302	Cox A24(G3)/1/Shanghai/CHN/1986(3C)	G3	Reference strain
China	Taiwan	1988	D10324	Cox A24/2/Kaohsiung/Taiwan/1988(3C)	G3	
China	Taiwan	1988	D10323	Cox A24/1/Kaohsiung/Taiwan/1988(3C)	G3	
China	Taiwan	1988	D13275	Cox A24/151/Taiwan/1988(3C)	G3	
China	Taiwan	1988	D13279	Cox A24/E-340/Taiwan/1988(3C)	G3	
China	Taiwan	1988	D13278	Cox A24/590/Taiwan/1988(3C)	G3	
China	Hong Kong	1988	D10321	Cox A24(G3)/2/Hong Kong/1988 (3C)	G3	Reference strain
China	Taiwan	1989	D13285	Cox A24/865/Taiwan/1989(3C)	G3	
China	Taiwan	1989	D13284	Cox A24/804/Taiwan/1989(3C)	G3	
China	Taiwan	1989	D13283	Cox A24/740/Taiwan/1989(3C)	G3	
China	Taiwan	1989	D13282	Cox A24/E-398/Taiwan/1989(3C)	G3	

China	Taiwan	1989	D13281	Cox A24/722/Taiwan/1989(3C)	G3	
China	Taiwan	1989	D13280	Cox A24/380/Taiwan/1989(3C)	G3	
China	Taiwan	1990	AB008476	Cox A24/62/Taiwan/1990(3C)	G3	
China	Taiwan	1993	AB008485	Cox A24/46/Taiwan/1993(3C)	G3	
China	Taiwan	1993	AB008484	Cox A24/26/Taiwan/1993(3C)	G3	
China	Taiwan	1993	AB008483	Cox A24/7/Taiwan/1993(3C)	G3	
China	Taiwan	1994	AB008482	Cox A24/88/Taiwan/1994(3C)	G3	
China	Taiwan	1994	AB008481	Cox A24/72/Taiwan/1994(3C)	G3	
China	Taiwan	1994	AB008480	Cox A24/66/Taiwan/1994(3C)	G3	
China	Taiwan	1994	AB008479	Cox A24/63/Taiwan/1994(3C)	G3	
China	Taiwan	1994	AB008478	Cox A24/62/Taiwan/1994(3C)	G3	
China	Taiwan	1994	AB008477	Cox A24/42/Taiwan/1994(3C)	G3	
China	Taiwan	2000	DQ472141	Cox A24(G4-c1)/K1350/Taiwan/2000(3C)	G4-c1	Reference strain
China	Zhejiang	2002	AY876912	Cox A24/3/Ningbo/CHN/2002(3C)	G4-c2	
Brazil	Unknown	2004	GU983234	Cox A24(G4-c2)/15/Brazil/2004(3C)	G4-c2	Reference strain
Brazil	Unknown	2005	GU983240	Cox A24(G4-c2)/182/Brazil/2005(3C)	G4-c2	Reference strain
China	Yunnan	2007	GQ229410	Cox A24/SF18/Yunnan/CHN/2007(3C)	G4-c3	
China	Yunnan	2007	GQ229409	Cox A24/ SF17/Yunnan/CHN/2007(3C)	G4-c3	
China	Yunnan	2007	GQ229408	Cox A24/SF12/Yunnan/CHN/2007(3C)	G4-c3	
China	Yunnan	2007	GQ229407	Cox A24/SF10/Yunnan/CHN/2007(3C)	G4-c3	
China	Yunnan	2007	GQ229406	Cox A24/SF9/Yunnan/CHN/2007(3C)	G4-c3	
China	Yunnan	2007	GQ229405	Cox A24/SF6/Yunnan/CHN/2007(3C)	G4-c3	
China	Yunnan	2007	GQ229404	Cox A24/SJ9/Yunnan/CHN/2007(3C)	G4-c3	
China	Yunnan	2007	GQ229403	Cox A24/SJ7/Yunnan/CHN/2007(3C)	G4-c3	
China	Yunnan	2007	GQ229402	Cox A24/SJ5/Yunnan/CHN/2007(3C)	G4-c3	
China	Yunnan	2007	GQ229401	Cox A24/ SJ4/Yunnan/CHN/2007(3C)	G4-c3	
China	Yunnan	2007	GQ229400	Cox A24/SJ3/Yunnan/CHN/2007(3C)	G3	
China	Guangdong	2007	EU409823	Cox A24/332/Guangdong/CHN/2007(3C)	G4-c3	
China	Guangdong	2007	EU409822	Cox A24/333/Guangdong/CHN/2007(3C)	G4-c3	
China	Guangdong	2007	EU409821	Cox A24/334/Guangdong/CHN/2007(3C)	G4-c3	
China	Guangdong	2007	EU409820	Cox A24/385/Guangdong/CHN/2007(3C)	G4-c3	
China	Guangdong	2007	EU409819	Cox A24/388/Guangdong/CHN/2007(3C)	G4-c3	

China	Guangdong	2007	EU409818	Cox A24/391/Guangdong/CHN/2007(3C)	G4-c3	
China	Guangdong	2007	EU409817	Cox A24/464/Guangdong/CHN/2007(3C)	G4-c3	
China	Guangdong	2007	EU409816	Cox A24/376/Guangdong/CHN/2007(3C)	G4-c3	
China	Guangdong	2007	EU409815	Cox A24/380/Guangdong/CHN/2007(3C)	G4-c3	
China	Guangdong	2007	EU409814	Cox A24/381/Guangdong/CHN/2007(3C)	G4-c3	
China	Guangdong	2007	EU409813	Cox A24/383/Guangdong/CHN/2007(3C)	G4-c3	
China	Zhejiang	2007	GQ429282	Cox A24/3/Zhejiang/CHN/2007(3C)	G4-c3	
China	Zhejiang	2007	GQ429281	Cox A24/2/Zhejiang/CHN/2013(3C)	G4-c2	
China	Guangdong	2007	EU409823	Cox A24(G4-c3)/223/Guangdong/CHN/2007(3C)	G4-c3	Reference strain
China	Zhejiang	2008	GQ429285	Cox A24/26/Zhejiang/CHN/2008(3C)	G4-c3	
China	Zhejiang	2008	GQ429284	Cox A24/20/Zhejiang/CHN/2008(3C)	G4-c3	
China	Zhejiang	2008	GQ429283	Cox A24/13/Zhejiang/CHN/2008(3C)	G4-c3	
Brazil	Unknown	2009	GU983242	Cox A24(G4-c4)/10/Brazil/2009(3C)	G4-c4	Reference strain
China	Guangdong	2010	JF742575	Cox A24/3/Guangdong/CHN/2010(3C)	G4-5a	
China	Guangdong	2010	JF742574	Cox A24/1/Guangdong /CHN/2010(3C)	G4-5a	
China	Guangdong	2010	JF742573	Cox A24/46/Guangdong/CHN/2010(3C)	G4-5a	
China	Guangdong	2010	JF742572	Cox A24/42/Guangdong/CHN/2010(3C)	G4-5a	
China	Guangdong	2010	JF742571	Cox A24/41/Guangdong/CHN/2010(3C)	G4-5a	
China	Guangdong	2010	JF742570	Cox A24/40/Guangdong/CHN/2010(3C)	G4-5a	
China	Guangdong	2010	JF742569	Cox A24/39/Guangdong/CHN/2010(3C)	G4-5a	
China	Guangdong	2010	JF742568	Cox A24/38/Guangdong/CHN/2010(3C)	G4-5a	
China	Guangdong	2010	JF742567	Cox A24/36/Guangdong/CHN/2010(3C)	G4-5a	
China	Guangdong	2010	JF742566	Cox A24/35/Guangdong/CHN/2010(3C)	G4-5a	
China	Guangdong	2010	JF742565	Cox A24/34/Guangdong/CHN/2010(3C)	G4-5a	
China	Guangdong	2010	JF742564	Cox A24/33/Guangdong/CHN/2010(3C)	G4-5a	
China	Guangdong	2010	JF742563	Cox A24/32/Guangdong/CHN/2010(3C)	G4-5a	
China	Guangdong	2010	JF742562	Cox A24/29/Guangdong/CHN/2010(3C)	G4-5a	
China	Guangdong	2010	JF742561	Cox A24/27/Guangdong/CHN/2010(3C)	G4-5a	
China	Guangdong	2010	JF742560	Cox A24/26/Guangdong/CHN/2010(3C)	G4-5a	
China	Jiangsu	2010	JN788288	Cox A24/P47/Jiangsu /CHN/2010(3C)	G4-5b	
China	Jiangsu	2010	JN788287	Cox A24/P46/Jiangsu /CHN/2010(3C)	G4-5b	
China	Jiangsu	2010	JN788286	Cox A24/P45/Jiangsu /CHN/2010(3C)	G4-5b	

China	Jiangsu	2010	JN788285	Cox A24/P41/Jiangsu /CHN/2010(3C)	G4-5a	
China	Jiangsu	2010	JN788284	Cox A24/P29/Jiangsu /CHN/2010(3C)	G4-5a	
China	Jiangsu	2010	JN788283	Cox A24/P19/Jiangsu /CHN/2010(3C)	G4-5a	
China	Jiangsu	2010	JN788282	Cox A24/P18/Jiangsu/CHN/2010(3C)	G4-5b	
China	Jiangsu	2010	JN788281	Cox A24/P17/Jiangsu/CHN/2010(3C)	G4-5b	
China	Jiangsu	2010	JN788280	Cox A24/P16/Jiangsu/CHN/2010(3C)	G4-5b	
China	Jiangsu	2010	JN788279	Cox A24/P15/Jiangsu/CHN/2010(3C)	G4-5b	
China	Jiangsu	2010	JN788278	Cox A24/P13/Jiangsu/CHN/2010(3C)	G4-5b	
China	Jiangsu	2010	JN788277	Cox A24/P12/Jiangsu/CHN/2010(3C)	G4-5b	
China	Jiangsu	2010	JN788276	Cox A24/P11/Jiangsu/CHN/2010(3C)	G4-5b	
China	Jiangsu	2010	JN788275	Cox A24/P10/Jiangsu/CHN/2010(3C)	G4-5b	
China	Jiangsu	2010	JN788274	Cox A24/P9/Jiangsu/CHN/2010(3C)	G4-5b	
China	Jiangsu	2010	JN788273	Cox A24/P8/Jiangsu/CHN/2010(3C)	G4-5b	
China	Jiangsu	2010	JN788272	Cox A24/P6/Jiangsu/CHN/2010(3C)	G4-5b	
China	Jiangsu	2010	JN788271	Cox A24/P4/Jiangsu/CHN/2010(3C)	G4-5b	
China	Jiangsu	2010	JN788270	Cox A24/P3/Jiangsu/CHN/2010(3C)	G4-5b	
China	Jiangsu	2010	JN788269	Cox A24/P1/Jiangsu/CHN/2010(3C)	G4-5b	
China	Zhejiang	2010	HQ699673	Cox A24/12/Zhejiang/CHN/2010(3C)	G4-5b	
China	Zhejiang	2010	HQ699672	Cox A24/10/Zhejiang/CHN/2010(3C)	G4-5b	
China	Zhejiang	2010	HQ699671	Cox A24/5/Zhejiang/CHN/2010(3C)	G4-5b	
China	Zhejiang	2010	HQ699670	Cox A24/4/Zhejiang/CHN/2010(3C)	G4-5b	
China	Jiangsu	2010	JN788283	Cox A24(G4-5a)/P19/Jiangsu/CHN/2010(3C)	G4-5a	Reference strain
China	Jiangsu	2010	JN788278	Cox A24(G4-5b)/P13/Jiangsu/CHN/2010(3C)	G4-5b	Reference strain

Supplementary table 2. Accession numbers of VP1 gene from Human coxsackievirus A24 isolate used in this study isolated in China (N=96)

Country	Area	Collect year	Accession number	Strain Name	Genotype	Notes
Brazil	Unknown	1987	EF015037	Cox A24(G2)/10628/Brazil/1987(VP1)	G2	Reference strain
Brazil	Unknown	1987	EF015038	Cox A24(G2)/10629/Brazil/1987(VP1)	G2	Reference strain
Brazil	Unknown	1987	GU983190	Cox A24(G3)/BR-PA-1/Brazil/1987(VP1)	G3	Reference strain
Singapore		1970	D90457	Cox A24(G1)/EH24/70/Singapore/1970(VP1)	G1	Reference strain
Taiwan	Taiwan	2000	AB473433	Cox A24(G4-c1)/K1351/Taiwan/2000(VP1)	G4-c1	Reference strain
Taiwan	Taiwan	2001	AB473434	Cox A24(G4-c1)/K0676/Taiwan/2001(VP1)	G4-c1	Reference strain
Spain	Unknown	2004	EU162078	Cox A24(G4-c2)/4192/Spain/2004(VP1)	G4-c2	Reference strain
Brazil	Unknown	2004	GU983197	Cox A24(G4-c2)/15/Brazil/2004(VP1)	G4-c2	Reference strain
India	Unknown	2007	GU477583	Cox A24(G4-c3)/M133/India/2007(VP1)	G4-c3	Reference strain
India	Unknown	2007	GU477576	Cox A24(G4-c4)/M110/India/2007(VP1)	G4-c4	Reference strain
Japan	Ishigaki	2011	AB769164	Cox A24(G4-c5a)/29/Japan/2011(VP1)	G4-c5a	Reference strain
CHN	Zhejiang	2010	HQ699669	Cox A24(G4-c5b)/12/Zhejiang/CHN/2010(VP1	G4-c5b	Reference strain
China	Guangdong	2010	JX154991	Cox A24/1101/Guangdong/CHN/2010(VP1)	G4-5a	
China	Guangdong	2010	JF742595	Cox A24/03/Guangdong/CHN/2010(VP1)	G4-5a	
China	Guangdong	2010	JF742594	Cox A24/01/Guangdong/CHN/2010(VP1)	G4-5a	
China	Guangdong	2010	JF742593	Cox A24/46/Guangdong/CHN/2010(VP1)	G4-5a	
China	Guangdong	2010	JF742592	Cox A24/42/Guangdong/CHN/2010(VP1)	G4-5a	
China	Guangdong	2010	JF742591	Cox A24/41/Guangdong/CHN/2010(VP1)	G4-5a	
China	Guangdong	2010	JF742590	Cox A24/40/Guangdong/CHN/2010(VP1)	G4-5a	
China	Guangdong	2010	JF742589	Cox A24/39/Guangdong/CHN/2010(VP1)	G4-5a	
China	Guangdong	2010	JF742587	Cox A24/36/Guangdong/CHN/2010(VP1)	G4-5a	
China	Guangdong	2010	JF742586	Cox A24/35/Guangdong/CHN/2010(VP1)	G4-5a	
China	Guangdong	2010	JF742585	Cox A24/34/Guangdong/CHN/2010(VP1)	G4-5a	
China	Guangdong	2010	JF742583	Cox A24/32/Guangdong/CHN/2010(VP1)	G4-5a	
China	Guangdong	2010	JF742582	Cox A24/29/Guangdong/CHN/2010(VP1)	G4-5a	
China	Guangdong	2010	JF742581	Cox A24/27/Guangdong/CHN/2010(VP1)	G4-5a	
China	Guangdong	2010	JF742580	Cox A24/26/Guangdong/CHN/2010(VP1)	G4-5a	
China	Guangdong	2010	JF742588	Cox A24/38/Guangdong/CHN/2010(VP1)	G4-5a	

China	Guangdong	2010	JF742584	Cox A24/33/Guangdong/CHN/2010(VP1)	G4-5a	
China	Jiangsu	2010	JN788305	Cox A24/P41/Jiangsu/CHN/2010(VP1)	G4-5a	
China	Jiangsu	2010	JN788304	Cox A24/P29/Jiangsu/CHN/2010(VP1)	G4-5a	
China	Jiangsu	2010	JN788303	Cox A24/P19/Jiangsu/CHN/2010(VP1)	G4-5a	
China	Shandong	2010	JQ728989	Cox A24/QD019/Shandong/CHN/2010(VP1)	G4-5a	
China	Shandong	2010	JQ728988	Cox A24/QD017/Shandong/CHN/2010(VP1)	G4-5a	
China	Shandong	2010	JQ728987	Cox A24/QD016/Shandong/CHN/2010(VP1)	G4-5a	
China	Shandong	2010	JQ728986	Cox A24/QD015/Shandong/CHN/2010(VP1)	G4-5a	
China	Shandong	2010	JQ728985	Cox A24/QD012/Shandong/CHN/2010(VP1)	G4-5a	
China	Shandong	2010	JQ728984	Cox A24/LY007/Shandong/CHN/2010(VP1)	G4-5a	
China	Shandong	2010	JQ728983	Cox A24/LY004/Shandong/CHN/2010(VP1)	G4-5a	
China	Shandong	2010	JQ728982	Cox A24/LY003/Shandong/CHN/2010(VP1)	G4-5a	
China	Shandong	2010	JQ728981	Cox A24/LY002/Shandong/CHN/2010(VP1)	G4-5a	
China	Shandong	2010	JQ728980	Cox A24/LY001/Shandong/CHN/2010(VP1)	G4-5a	
China	Yunnan	2011	AB813038	Cox A24/487-1/Yunnan/CHN/2011(VP1)	G4-5a	
China	Yunnan	2011	AB813037	Cox A24/197-1/Yunnan/CHN/2011(VP1)	G4-5a	
China	Jiangsu	2010	JN788308	Cox A24/P47/Jiangsu/CHN/2010(VP1)	G4-5b	
China	Jiangsu	2010	JN788307	Cox A24/P46/Jiangsu/CHN/2010(VP1)	G4-5b	
China	Jiangsu	2010	JN788306	Cox A24/P45/Jiangsu/CHN/2010(VP1	G4-5b	
China	Jiangsu	2010	JN788302	Cox A24/P18/Jiangsu/CHN/2010(VP1)	G4-5b	
China	Jiangsu	2010	JN788301	Cox A24/P17/Jiangsu/CHN/2010(VP1)	G4-5b	
China	Jiangsu	2010	JN788300	Cox A24/P16/Jiangsu/CHN/2010(VP1)	G4-5b	
China	Jiangsu	2010	JN788299	Cox A24/P15/Jiangsu/CHN/2010(VP1)	G4-5b	
China	Jiangsu	2010	JN788298	Cox A24/P13/Jiangsu/CHN/2010(VP1)	G4-5b	
China	Jiangsu	2010	JN788297	Cox A24/P12/Jiangsu/CHN/2010(VP1)	G4-5b	
China	Jiangsu	2010	JN788296	Cox A24/P11/Jiangsu/CHN/2010(VP1)	G4-5b	
China	Jiangsu	2010	JN788295	Cox A24/P10/Jiangsu/CHN/2010(VP1)	G4-5b	
China	Jiangsu	2010	JN788294	Cox A24/P9/Jiangsu/CHN/2010(VP1)	G4-5b	
China	Jiangsu	2010	JN788293	Cox A24/P8/Jiangsu/CHN/2010(VP1)	G4-5b	
China	Jiangsu	2010	JN788292	Cox A24/P6/Jiangsu/CHN/2010(VP1)	G4-5b	
China	Jiangsu	2010	JN788291	Cox A24/P4/Jiangsu/CHN/2010(VP1)	G4-5b	
China	Jiangsu	2010	JN788290	Cox A24/P3/Jiangsu/CHN/2010(VP1)	G4-5b	

China	Jiangsu	2010	JN788289	Cox A24/P1/Jiangsu/CHN/2010(VP1)	G4-5b	
China	Shandong	2003	GQ329725	Cox A24/03353/Shandong/CHN/2003(VP1)	G4-c2	
China	Guangdong	2007	EU391662	Cox A24/388/Guangdong/CHN/2007(VP1)	G4-c3	
China	Guangdong	2010	JX181930	Cox A24/025/Guangdong/CHN/2010(VP1)	G4-c3	
China	Shandong	1994	GU906789	Cox A24/94335/Shandong/CHN/1994(VP1)	G4-c3	
China	Shandong	1999	GQ329726	Cox A24/99053/Shandong/CHN/1999(VP1)	G4-c3	
China	Shandong	2009	GU906788	Cox A24/09088/Shandong/CHN/2009(VP1)	G4-c3	
China	Yunnan	1997	AB268298	Cox A24/169/Yunnan/CHN/1997(VP1)	G4-c3	
China	Yunnan	1998	AB268304	Cox A24/8/Yunnan/CHN/1998(VP1)	G4-c3	
China	Yunnan	1998	AB268296	Cox A24/136/Yunnan/CHN/1998(VP1)	G4-c3	
China	Yunnan	1999	AB268305	Cox A24/94/Yunnan/CHN/1999(VP1)	G4-c3	
China	Yunnan	1999	AB268303	Cox A24/7/Yunnan/CHN/1999(VP1)	G4-c3	
China	Yunnan	1999	AB268299	Cox A24/198/Yunnan/CHN/1999(VP1)	G4-c3	
China	Yunnan	1999	AB268297	Cox A24/162/Yunnan/CHN/1999(VP1)	G4-c3	
China	Yunnan	1999	AB268295	Cox A24/102/Yunnan/CHN/1999(VP1)	G4-c3	
China	Yunnan	2000	AB268302	Cox A24/46/Yunnan/CHN/2000(VP1)	G4-c3	
China	Yunnan	2000	AB268301	Cox A24/253/Yunnan/CHN/2000(VP1)	G4-c3	
China	Yunnan	2000	AB268300	Cox A24/233/Yunnan/CHN/2000(VP1)	G4-c3	
China	Yunnan	2007	EU596589	Cox A24/SF17/Yunnan/CHN/2007(VP1)	G4-c3	
China	Yunnan	2007	EU596588	Cox A24/SF12/Yunnan/CHN/2007(VP1)	G4-c3	
China	Yunnan	2007	EU596587	Cox A24/SF9/Yunnan/CHN/2007(VP1)	G4-c3	
China	Yunnan	2007	EU596586	Cox A24/SF6/Yunnan/CHN/2007(VP1)	G4-c3	
China	Yunnan	2007	EU596584	Cox A24/SJ7/Yunnan/CHN/2007(VP1)	G4-c3	
China	Yunnan	2007	EU596583	Cox A24/SJ5/Yunnan/CHN/2007(VP1)	G4-c3	
China	Yunnan	2007	EU596582	Cox A24/SJ4/Yunnan/CHN/2007(VP1)	G4-c3	
China	Yunnan	2007	EU596581	Cox A24/SJ3/Yunnan/CHN/2007(VP1)	G4-c3	
China	Yunnan	2007	GQ229399	Cox A24/SF18/Yunnan/CHN/2007(VP1)	G4-c3	
China	Yunnan	2007	GQ229398	Cox A24/SF10/Yunnan/CHN/2007(VP1)	G4-c3	
China	Yunnan	2012	AB759898	Cox A24/ZK-26/Yunnan/CHN/2012(VP1)	G4-c3	
China	Zhejiang	2007	GQ429279	Cox A24/03/Zhejiang/CHN/2007(VP1)	G4-c3	
China	Zhejiang	2008	GQ429288	Cox A24/26/Zhejiang/CHN/2008(VP1)	G4-c3	
China	Zhejiang	2008	GQ429286	Cox A24/15/Zhejiang/CHN/2008(VP1)	G4-c3	

China	Zhejiang	2010	HQ699669	Cox A24/12/Zhejiang/CHN/2010(VP1)	G4-c5b	
China	Zhejiang	2010	HQ699668	Cox A24/10/Zhejiang/CHN/2010(VP1)	G4-c5b	
China	Zhejiang	2010	HQ699667	Cox A24/05/Zhejiang/CHN/2010(VP1)	G4-c5b	
China	Zhejiang	2010	HQ699666	Cox A24/04/Zhejiang/CHN/2010(VP1)	G4-c5b	
